# Feasibility and safety profile of intranasal third-party allogeneic CD45RO^+^/RA^−^ memory T cells: a phase I clinical trial (vRELEASE-I NCT06699758)

**DOI:** 10.3389/fmed.2026.1832888

**Published:** 2026-07-03

**Authors:** Karima Al-Akioui-Sanz, Fiorela C. Dueñas Lopez, Javier Guijarro-Eguinoa, Arturo Gómez Lopez De Las Huertas, Paula Lázaro, Mercedes Gasior, Lidia Pertíñez, Laura Clares Villa, Carmen Mestre Durán, Alberto M. Borobia, Antonio Balas, Cristina Calvo, Antonio Pérez-Martínez

**Affiliations:** 1 CIBERER-ISCIII, IdiPAZ-CNIO Translational Research Unit in Pediatric Hemato-Oncology, La Paz University Hospital Research Institute-Spanish National Cancer Center, Madrid, Spain; 2 Department of Clinical Pharmacology, La Paz University Hospital (IdiPaz), Madrid, Spain; 3 Bone Marrow and Cell Therapy Unit, Hematology Department La Paz University Hospital, Madrid, Spain; 4 Department of Pharmacology, School of Medicine, Autonomous University of Madrid, Spain; 5 HLA Typing, Centro de Transfusiones Comunidad de Madrid, Madrid, Spain; 6 Department of Pediatric Infectious Diseases, La Paz University Hospital, Madrid, Spain; 7 CIBERINFEC-ISCIII, Madrid, Spain; 8 Department of Pediatric, Autonomous University of Madrid, Spain; 9 Department of Pediatric Hemato-Oncology, La Paz University Hospital, Madrid, Spain

**Keywords:** adoptive T-Cell therapy, intranasal administration, mucosal immunity, off-the-shelf cell-based immunotherapy, respiratory viral infections, third-party CD45RO^+^/RA^−^ memory T cells

## Abstract

**Background:**

Respiratory viral infections remain a major global health challenge, particularly in immunocompromised populations. Although adoptive cell therapies using virus-experienced memory T lymphocytes have demonstrated safety and effectiveness when administered intravenously, the safety profile and feasibility of intranasal delivery of immune effector cells in humans was not previously assessed.

**Methods:**

vRELEASE I (NCT06699758) represents a phase I, single-center, open-label, dose-escalation study designed to evaluate the safety profile and feasibility of intranasal administration of off-the-shelf, allogeneic CD45RO^+^/RA^−^ memory T lymphocytes derived from a third-party male donor in healthy female adult volunteers. The study employed a conventional 3+3 dose-escalation design, evaluating three dose levels (1 × 10^6^, 5 × 10^6^, and 10 × 10^6^ cells per dose). Each participant received three intranasal administrations at 2-h intervals. Safety assessments included monitoring for adverse events and dose-limiting toxicities (DTLs). The local persistence of donor-derived cells in the nasal mucosa was evaluated using fluorescence *in situ* hybridization (FISH).

**Results:**

Intranasal administration was completed as planned in all nine treated participants. No treatment-emergent adverse events were observed in any cohort during the seven-day safety follow-up period. The highest planned dose, 10 × 10^6^ cells per administration, was delivered to three participants without DLTs during short-term follow-up. Donor-derived male cells were not detected above the assay detection threshold in evaluable nasal mucosa samples by Y-chromosome FISH.

**Conclusion:**

In this first-in-human, single-center phase I dose-escalation study, intranasal administration of third-party, ready-to-use, allogeneic CD45RO^+^/RA^−^ memory T lymphocytes was well tolerated. No treatment-emergent adverse events or DTLs occurred during the seven-day follow-up of nine healthy adult female volunteers, confirming the operational feasibility of the planned dose levels. These preliminary findings support further monitored evaluation in clinically relevant populations, though longer follow-up remains necessary to assess delayed immunological or late adverse events.

## 1 Introduction

Acute respiratory viral infections caused by Respiratory Syncytial Virus (RSV), SARS-CoV-2, and Influenza Virus impose a heavy burden on global healthcare systems, causing recurrent seasonal epidemic waves. This impact is most severe among vulnerable groups, including young children, older adults, and individuals with underlying comorbidities (1–5). According to recent World Health Organization (WHO) data, RSV alone causes approximately 3.6 million hospitalizations and over 100,000 deaths annually in children under five years of age. Concurrently, global SARS-CoV-2 transmission persists with continuous morbidity, and recent surveillance data from the Global Influenza Surveillance and Response System (GISRS) show heightened influenza activity across multiple regions (3–5).

Current preventive strategies, including maternal vaccines, long-acting monoclonal antibodies, and seasonal vaccines, primarily offer temporary or passive protection, leaving a critical therapeutic gap once severe disease is established (6–8). Crucially, severe outcomes are driven by dysregulated host immune responses rather than viral burden alone (9–11).

Protective immunity requires coordinated mucosal T-cell responses, yet these viruses frequently cause T-cell exhaustion, dysregulation, or immune evasion (11–13). Supporting and restoring effective antiviral cellular immunity represents an innovative therapeutic approach to control infection and prevent severe disease when humoral immunity fails (12, 14).

Adoptive cell therapy using virus-experienced memory T lymphocytes has therefore emerged as a promising strategy to restore antiviral immunity in immunocompromised patients (15). Third-party, ready-to-use CD45RO^+^/RA^−^ cells containing pathogen-specific memory T lymphocytes and characterized by low alloreactivity, have been safely administered intravenously in multiple clinical settings, including hematopoietic stem cell transplantation and the treatment of severe viral infections (14–17). In particular, a Phase I/II dose-escalation clinical trial demonstrated the safety and feasibility of intravenous infusion of CD45RO^+^/RA^−^ memory T lymphocytes in patients with severe SARS-CoV-2 infection (15). These findings were subsequently supported by a randomized Phase II clinical trial, which showed immune reconstitution and improved clinical outcomes in patients receiving memory T-cell therapy in addition to standard of care (14). Furthermore, other cell-based approaches support the relevance of using cell therapy to treat respiratory infections (18–20).

Intranasal cell delivery directly targets the respiratory mucosa where infections begin, offering a non-invasive approach that maximizes local immunity while minimizing systemic side effects (21–24). While preclinical data and recent advancements, such as intranasal liposomal vaccines (25), demonstrate effective mucosal distribution and safety, the intranasal administration of differentiated immune effector cells has never been evaluated in humans.

The vRELEASE I (NCT06699758) clinical trial was designed as a first-in-human, proof-of-concept Phase I study to assess the safety porfile and feasibility of intranasal administration of third-party CD45RO^+^/RA^−^ memory T lymphocytes. By enrolling healthy adult female participants and employing a conservative 3+3 dose-escalation design in accordance with the International Council for Harmonization Good Clinical Practice (ICH-GCP) guideline, ICH E6(R2) (26) guidelines, the study was not designed to assess antiviral efficacy but to establish the maximum tolerated dose while carefully characterizing local and systemic safety signals. In addition, the trial explores the local persistence of donor-derived memory T lymphocytes in the nasal mucosa using sex-mismatched cell tracking, providing direct insight into biodistribution following intranasal delivery.

Establishing the safety of this novel route of administration represents a critical translational step toward the development of locally targeted immunotherapies for respiratory viral infections, which continue to pose a major unmet medical need worldwide (1, 2, 27). The findings of this study provide foundation for future clinical trials evaluating the therapeutic potential of intranasally delivered ready-to-use memory T lymphocytes in vulnerable populations affected by severe respiratory diseases (14).

## 2 Materials and methods

### 2.1 Study design

vRELEASE I constitutes a Phase I, single-center, open-label, non-randomized, dose-escalation clinical trial to asses the safety and feasibility of intranasal administration of third-party CD45RO^+^/RA^−^ memory T lymphocytes derived from a healthy male donor in healthy adult female volunteers. The study adhered to a standard 3+3 dose-escalation design and was conducted as a proof-of-concept investigation devoid of therapeutic intent. The trial followed the principles of the Declaration of Helsinki, the ICH-GCP guidelines, and European regulatory requirements.

### 2.2 Study population

Healthy adult female participants aged 18 to 55 years were eligible for inclusion. Restriction to female participants enabled sex-mismatched donor cell tracking using Y-chromosome-based detection methods. All participants were required to be in good general health, operationally defined in our clinical trials unit as the absence of chronic medical conditions, the absence of ongoing medical follow-up, and the absence of chronic or regular medication use. Key exclusion criteria included recent or ongoing respiratory infections, pregnancy or breastfeeding status, known immunodeficiency, history of chronic illnesses, current engagement in another clinical trial or participation within the preceding three months, and any condition that might compromise protocol adherence or safety assessments. The absence of recent or ongoing respiratory infection was verified through a directed clinical interview and physical examination performed both at the screening visit and on the day of administration. Participants were excluded if they reported any of the following symptoms within the previous 7 days: fever, chills, myalgia, rhinorrhea, sore throat, cough, dyspnea, headache, nausea, vomiting or diarrhea. No systematic microbiological testing for respiratory pathogens was performed, in line with standard practice for first-in-human safety studies in healthy volunteers. Written informed consent was obtained from all participants before any study-related procedures.

### 2.3 Investigational product

The investigational product comprised a suspension of allogeneic third-party CD45RO^+^/RA^−^ memory T lymphocytes, isolated from peripheral blood mononuclear cells obtained from a healthy adult male donor. These cells were derived from excess material collected during routine leukapheresis procedures, with donor's full positive clinical evaluation and consent allowing secondary research utilization. HLA typing of healthy donor and volunteers (V1 to V9) was performed at the Community of Madrid Transfusion Center (Madrid, Spain) on two independent samples by sequence-specific oligonucleotide and next-generation sequencing. In all cases, we found partial HLA-match, with at least one compatible allele (Supplementary Table S1).

**Figure 1:**
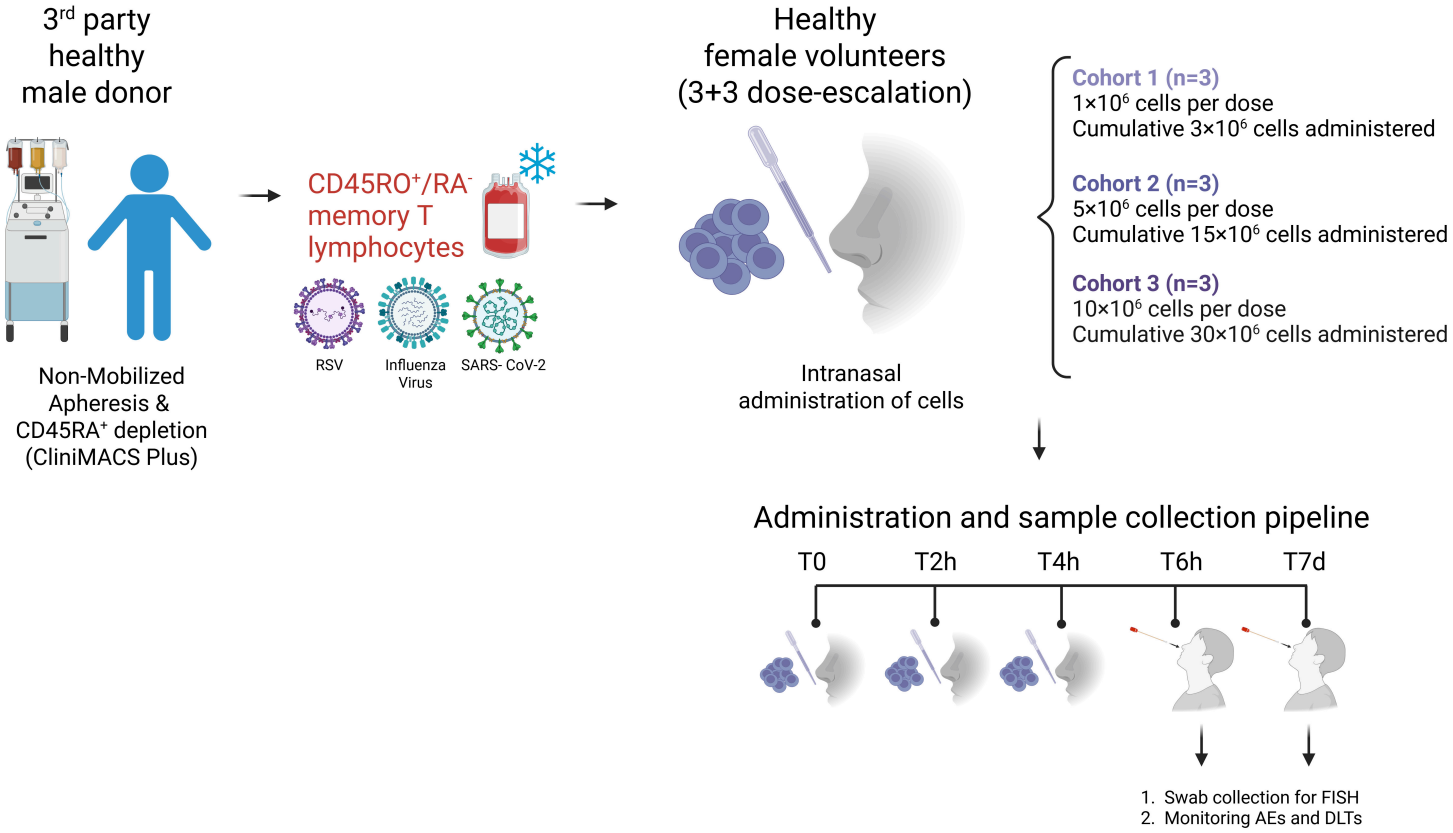
Scheme of the procedure. A male healthy donor received a non-mobilized apheresis, followed by a CD45RA^+^ cells depletion. CD45RO^+^/RA^−^ fraction obtained was frozen in cryopreservation bags and stored in N_2_ until administration. Before administration, cells were dry-thawed, washed, resuspended in saline solution and then applied in the female volunteers' noses by a sterile Pasteur pipette. The study employed a standard 3+3 dose-escalation design with three sequential dose cohorts. Each participant received three intranasal administrations at 2-h intervals (T0, T2, and T4), resulting in a cumulative dose per participant. Adverse events (AEs) and dose-limiting toxicities (DLTs) were assessed, and nasal mucosal samples were collected by swabs 2 h after the last administration time point (T6), and 7 days later (T7), to assess local persistence of donor-derived cells within the nasal mucosa by fluorescence in situ hybridization (FISH). Created with BioRender.com.

CD45RO^+^/RA^−^ memory T lymphocytes were obtained performing a non-mobilized apheresis at the Bone Marrow Transplantation and Cell Therapy Unit of University Hospital La Paz (Madrid, Spain), in accordance with routine clinical protocols, followed by CD45RA^+^ cells depletion using a CliniMACS Plus cell separation system (Miltenyi Biotec). Before and after cellular processing, cell count was performed using an automated hematology analyzer (Cell-Dyn Emerald) and viability and functionality were assessed by flow cytometry and interferon-gamma (IFN-γ) response assay (16), demonstrating that the product met predefined release criteria and complied with the quality standards established by the unit for clinical use. In addition, microbiological cultures were obtained to confirm sterility. After processing, CD45RA depleted cells were frozen in cryopreservation bags using 50% Buffer + 45% autologous plasma + 5% DMSO and maintained in liquid N_2_ until administration. This procedure was assessed and validated previously in the unit. Cells were dry thawed immediately prior to administration using a thawing/warming System (Transmed-SAHARA TSC) and washed in sterile CliniMACS PBS-EDTA Buffer (Miltenyi Biotec). Then, cells were resuspended in 1.5 ml of saline solution, counted again by Trypan-blue staining, and refrigerated until direct intranasal administration (Figure 1). Functionality and immunophenotype after thawing and washing was evaluated again by interferon-gamma (IFN-γ) response assay and flow cytometry techniques (Supplementary Tables S2– S5).

### 2.4 Dose escalation and treatment administration

The study employed a standard 3+3 dose-escalation design with three sequential dose cohorts. Each participant received three intranasal administrations at 2-h intervals (T0, T2, and T4), resulting in a cumulative dose per participant. The timing and the three-dose schedule were selected to maximize mucosal exposure while ensuring a conservative safety profile, as well as cell viability for potential cell detection after administration.

The planned dose levels were:

- Cohort 1: cumulative 3 × 10^6^ cells administered over three 1 × 10^6^ cells per dose.
- Cohort 2: cumulative 15 × 10^6^ cells administered over three 5 × 10^6^ cells per dose.
- Cohort 3: cumulative 30 × 10^6^ cells administered over three 10 × 10^6^ cells per dose.

**Figure 2:**
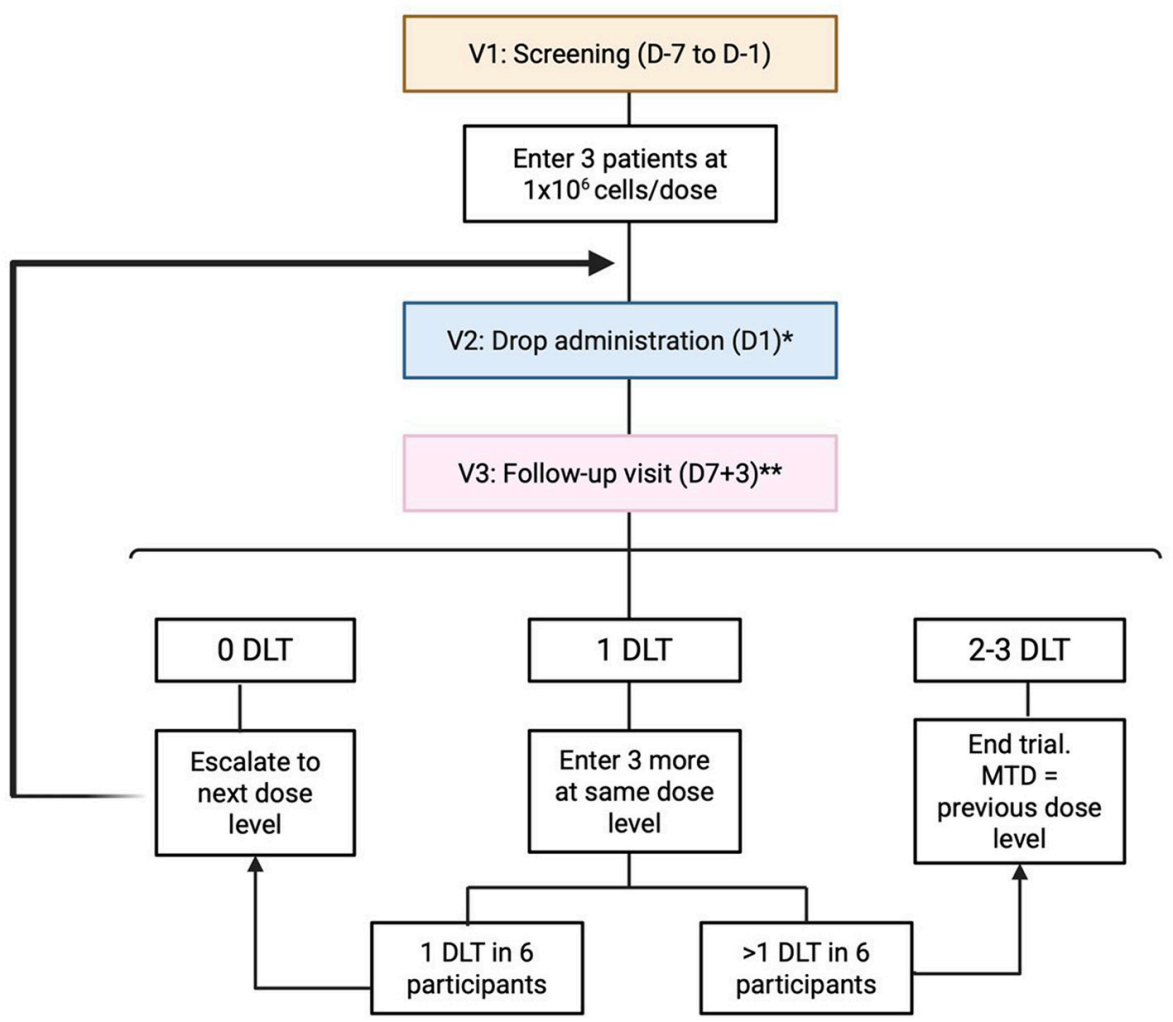
Administration-day schedule and follow-up in the 3+3 Design vRELEASE-I Phase I Clinical Trial.*On the administration day (Visit 2, V2, at day 1, D1), each participant received three intranasal doses of the investigational product at 2-h intervals (T0, T2 and T4) and was monitored on-site for systemic and local adverse events for a total of 6 h after the first administration, including at least 2 h after the last dose. Nasal mucosal samples were collected before the first administration and 2 h after the last administration. † A follow-up safety visit (Visit 3, V3) was performed on-site 7 days (D7) after administration (+3-day window) to assess adverse events and dose-limiting toxicities; an additional nasal sample was obtained at this visit, which marked the end of the study. DLT, dose-limiting toxicity; MTD, maximum tolerated dose. Created with BioRender.com.

The dose-escalation strategy was designed conservatively, taking into account prior intravenous clinical experience with third-party CD45RO^+^/RA^−^ memory T lymphocytes from our group, the first-in-human nature of the intranasal route for this cellular product, and the anatomical volume constraints of nasal administration. The selected dose levels were expressed both per intranasal administration and as cumulative dose per participant. Each administration was limited to approximately 0.5 mL, delivered as five drops per nostril, to promote local mucosal exposure while minimizing nasal runoff, swallowing of the product, or mechanical obstruction. The split-dose schedule, consisting of three administrations at 2-h intervals, was intended to maximize mucosal exposure under close clinical monitoring while maintaining a conservative safety profile. Participants remained under on-site medical supervision for a total of 6 h following the initial administration, including at least 2 h after the final dose. Dose escalation to the subsequent cohort was undertaken only after completion of safety evaluations for all individuals in the preceding cohort (Figure 2).

### 2.5 Safety assessments

The primary objective of the study was to assess safety and tolerability. Safety evaluations included the monitoring of adverse events (AEs), serious adverse events (SAEs), and dose-limiting toxicities (DLTs). DLTs were defined as any grade 3 or higher adverse event, according to the Common Terminology Criteria for Adverse Events (CTCAE) (28), version 5.0, considered probably or definitely related to the investigational product.

Vital signs, physical examinations, and clinical laboratory assessments (including hematology and biochemistry) were conducted at baseline, during the administration visit, and at the follow-up visit. Participants received active monitoring for acute reactions during the on-site observation period, and for adverse events throughout the 7-day follow-up period (+3-day window). Given the intranasal route of administration, local tolerability was specifically assessed at each administration time point (before and after every intranasal dose) and at the 7-day follow-up visit through an active, investigator-led directed questioning. Participants were asked about local symptoms potentially attributable to the intranasal procedure, including nasal pain, burning, itching, congestion, rhinorrhea, epistaxis, altered smell or taste, sore throat and general nasal discomfort. This assessment was complemented by on-site clinical monitoring, vital signs, and hematological and biochemical laboratory evaluations at baseline, on the administration day and at the follow-up visit. Nasal endoscopy and validated standardized mucosal irritation scoring scales were not included in the protocol, in line with the proof-of-concept nature of the study in healthy, non-symptomatic volunteers.

### 2.6 Assessment of local donor cell persistence

As a secondary exploratory objective, the study evaluated the local persistence of donor-derived cells in the nasal mucosa following intranasal administration of the investigational product. Nasal samples were collected by nasal swabs prior to administration and 2 h after the last administration time point.

The presence of donor-derived cells was assessed by fluorescence *in situ* hybridization (FISH) targeting the Y chromosome, enabling qualitative detection of male donor cells in female recipients. Nasal mucus samples were fixed using Carnoy's solution (methanol:acetic acid, 3:1). Cells were hybridized using an X/Y chromosome probe set (MetaSystems, D-5608-100-OG), which allows detection of sex chromosomes through centromeric fluorescence signals. Sample and probe were denatured at 75°C for 2 min, followed by overnight hybridization in a humidified chamber at 37°C. Post-hybridization washes, counterstaining, and mounting were performed in accordance with the manufacturer's instructions. DAPI/antifade solution (MetaSystems, D-0902-500-DA) was applied prior to microscopic analysis. Fluorescence microscopy was performed using a Leica fluorescence microscope. Signal evaluation was conducted qualitatively and manually (non-automated), in compliance with IVDR certification requirements for *in vitro* diagnostic procedures. For each sample, up to 50 interphase nuclei were analyzed when sufficient cellular material was available, following the ACMG technical standards. Samples with insufficient numbers of preserved/interpretable nuclei or lack of reliable hybridization signal were conservatively reported as not assessable.

### 2.7 Statistical analysis

A total of nine to eighteen participants were planned for enrolment. Safety and persistence outcomes were summarized using descriptive statistics. Continuous variables were described using median and range due to the small sample size, while categorical variables were reported as counts and percentages. All participants who received at least one dose of the investigational product were included in the safety analysis set.

### 2.8 Ethical considerations

The study protocol, informed consent documents, and all pertinent study materials were reviewed and approved prior to the commencement of the study by the Research Ethics Committee of La Paz University Hospital as well as the Ministry of Health's National Organization of Transplant. The trial was prospectively registered at ClinicalTrials.gov under NCT06699758 before enrolment of the first participant. Confidentiality of participants and data protection measures were upheld in accordance with European data protection regulations.

## 3 Results

### 3.1 Participant disposition and baseline characteristics

**Figure 3:**
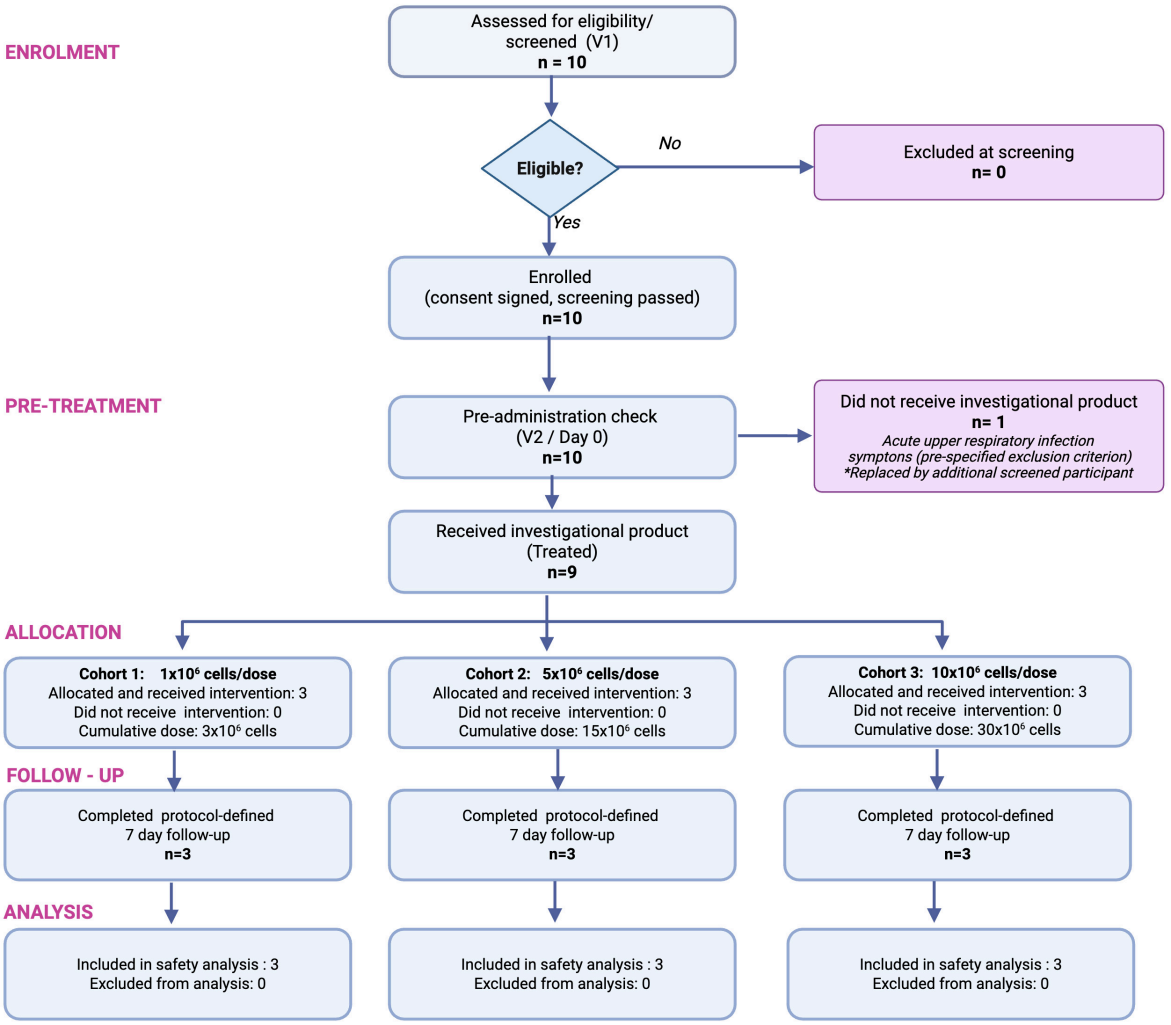
Participant flow diagram. This diagram, presented in accordance with CONSORT guidelines, delineates the number of participants screened, those excluded prior to treatment, enrolled individuals, those who received treatment, and those allocated to each dose cohort. It also details the participants who completed the protocol-specified seven-day follow-up and those included in the safety analysis population. Enrolment and treatment proceeded sequentially according to the 3+3 dose-escalation design: each cohort was screened, enrolled, treated, and followed up before screening of the subsequent cohort began. Dose escalation was contingent on completion of the safety evaluation of all participants in the preceding cohort. * One participant initially allocated in cohort 3 was excluded on the administration day (v2/Day0) due to symptoms compatible with an acute upper respiratory tract infection (pre-specified criterion). An additional volunteer was subsequently screened, enrolled, and assigned to cohort 3, where she received the investigational product as planned. Total participant screened and enrolled (consent signed, screened passed) = 10; total receiving investigational product = 9. V1, screened visit; V2, administration visit (Day 0); DLT, dose-limiting toxicity. No DLTs were observed at any dose level. Created with BioRender.com.

A total of ten participants were screened for eligibility, and all met the eligibility criteria at screening. All ten participants were subsequently enrolled in the study. One enrolled participant did not receive the investigational product because symptoms consistent with an acute upper respiratory tract infection were identified at the administration visit, corresponding to a pre-specified exclusion criterion. Consequently, nine participants received at least one intranasal administration and were included in the safety analysis population. All nine treated participants completed the protocol-defined seven-day follow-up visit. Participant disposition is shown in Figure 3.

Treated participants were sequentially allocated to three dose-escalation cohorts in accordance with the study protocol: Cohort 1 received 1 × 10^6^ cells per intranasal administration, Cohort 2 received 5 × 10^6^ cells per intranasal administration, and Cohort 3 received 10 × 10^6^ cells per intranasal administration. Each participant received three intranasal administrations at two-hour intervals, corresponding to cumulative doses of 3 × 10^6^, 15 × 10^6^, and 30 × 10^6^ cells, respectively. Each cohort included three treated participants (*n* = 3).

Baseline demographic and clinical characteristics are summarized in Table 1. The median age was 29 years [range (23–49)]. All treated participants were female and in good general health at study entry. No clinically significant abnormalities were detected in baseline laboratory assessments.

**Table 1:** Baseline demographic and clinical characteristics of the study population.

Variable	Dose cohort 1 - 1 x 10^6^ cells (*n* = 3)			Dose cohort 2 - 5 x 10^6^ cells (*n* = 3)			Dose cohort 3 - 10 x 10^6^ cells (*n* = 3)		
Patient number	#1	#2	#3	#4	#5	#6	#7	#8	#9^*^
Age (years)	27	23	49	30	25	30	42	28	29
Gender (M, F)	F	F	F	F	F	F	F	F	F
Weight (Kg)	54.4	64.0	58.2	42.9	82.7	59.1	44.6	54.4	58.9
Medication	None	None	None	None	None	None	None	None	None
Comorbidities	Pollen Allergy	None	Migraine	None	Migraine	None	None	None	None

Each participant (*n* = 3 per cohort, *n* = 9 total) received three intranasal administrations of the investigational product at the indicated per-dose level, administered 2 h apart (T0, T2 and T4).

^*^Participant #9 corresponds to the tenth enrolled subject; one participant was excluded prior to treatment due to symptoms consistent with a respiratory infection.

### 3.2 Treatment exposure and dose escalation

All treated participants received the three planned intranasal administrations of the investigational product at two-hour intervals corresponding to T0, T2, and T4. No missed administrations, dose reductions, delays, administration failures, or administration-related protocol deviations were reported. Dose exposure and protocol adherence are summarized in Table 2.

**Table 2:** Dose exposure and protocol adherence.

Cohort	Dose per intranasal administration	Number of administrations	Cumulative dose	Treated participants	Completed all planned administrations	Protocol deviations
Cohort 1	1 × 10^6^ cells	3	3 × 10^6^ cells	3	3/3	0/3
Cohort 2	5 × 10^6^ cells	3	15 × 10^6^ cells	3	3/3	0/3
Cohort 3	10 × 10^6^ cells	3	30 × 10^6^ cells	3	3/3	0/3
Total	**—**	**—**	**—**	**9**	**9/9**	**0/9**

Dose escalation proceeded according to the predefined 3+3 design. No DLTs were observed in any cohort during the seven-day safety follow-up period. The highest planned dose was 10 × 10^6^ cells per intranasal administration was achieved.

### 3.3 Safety and tolerability

Safety outcomes are reported across distinct components, including solicited local symptoms, unsolicited adverse events, serious adverse events, dose-limiting toxicities, clinical laboratory findings, vital signs, and pregnancy testing. FISH sample evaluability and donor-cell detection are reported separately as exploratory endpoints.

#### 3.3.1 Solicited local symptoms

Solicited local symptoms were assessed during the on-site observation period and at Day 7 follow-up visit using directed investigator-led questioning and clinical observation. No solicited local symptoms were reported in any treated participant at either assessment time point. Specifically, no nasal pain or discomfort, nasal irritation or burning, rhinorrhea, nasal congestion, sneezing, epistaxis, pruritus, local swelling, erythema, or other local reaction was reported. These findings reflect symptom-based local tolerability assessment and do not exclude subclinical mucosal changes. Solicited local symptoms are summarized in Table 3.

**Table 3:** Solicited local symptoms by assessment time point.

Solicited local symptom	On-site observation, 0–6 h	Day 7 follow-up
Nasal pain/discomfort	0/9	0/9
Nasal irritation/burning	0/9	0/9
Rhinorrhea	0/9	0/9
Nasal congestion	0/9	0/9
Sneezing	0/9	0/9
Epistaxis	0/9	0/9
Pruritus	0/9	0/9
Local swelling/erythema	0/9	0/9
Other local symptoms	0/9	0/9

Local symptoms were evaluated through systematic investigator-led questioning and clinical observation. Neither nasal endoscopy nor validated mucosal irritation scoring was conducted.

#### 3.3.2 Unsolicited adverse events, serious adverse events, and dose- limiting toxicities

No unsolicited adverse events were reported during the seven-day follow-up period. No treatment-emergent adverse events, treatment-related adverse events, serious adverse events, grade ≥3 adverse events, DLTs, or adverse events leading to discontinuation occurred in any cohort. These outcomes are summarized in Table 4. Based on these findings, the predefined criteria for dose escalation were met for all cohorts.

**Table 4:** Unsolicited adverse events, serious adverse events, and DLTs.

Safety outcome	Cohort 1 n/N	Cohort 2 n/N	Cohort 3 n/N	Total n/N
Any unsolicited adverse event	0/3	0/3	0/3	0/9
Treatment-emergent adverse event	0/3	0/3	0/3	0/9
Treatment-related adverse event	0/3	0/3	0/3	0/9
Grade ≥ 3 adverse event	0/3	0/3	0/3	0/9
Serious adverse event	0/3	0/3	0/3	0/9
Dose-limiting toxicity	0/3	0/3	0/3	0/9
Adverse event leading to discontinuation	0/3	0/3	0/3	0/9

#### 3.3.3 Clinical laboratory findings, vital signs, and pregnancy testing

No clinically significant changes in vital signs were observed in hematology, biochemistry, or vital sign assessments during the study. Vital signs were normal in all treated participants during the administration visit and at follow-up. Pregnancy tests were negative in all applicable participants at the protocol-defined assessment time point. Clinical laboratory findings, vital signs, and pregnancy testing are summarized in Table 5.

**Table 5:** Clinical laboratory findings, vital signs, and pregnancy testing were assessed.

	Dose cohort								
Variable	1 x 10^6^cells			5 x 10^6^ cells			10 x 10^6^ cells		
Patient number	#1	#2	#3	#4	#5	#6	#7	#8	#9
Visit 1									
Leukocytes (10^3^/uL)	6.44	5.36	7.15	6.25	4.78	4.36	4.13	5.1	6.84
Neutrophils (10^3^/uL)	3.61	3.24	4.82	3.63	2.63	1.74	2.21	2.41	4.38
Lymphocytes (10^3^/uL)	2.10	1.57	1.77	1.91	1.53	2.18	1.45	2.2	1.85
Visit 2									
Vital Signs	Normal	Normal	Normal	Normal	Normal	Normal	Normal	Normal	Normal
Pregnancy Test (WOBC)	Negative	Negative	Negative	Negative	Negative	Negative	Negative	Negative	Negative
Visit 3									
Leukocytes (10^3^/uL)	6.51	9.28	5.79	8.22	4.93	5.18	3.56	6.4	6.42
Neutrophils (10^3^/uL)	3.61	6.1	3.31	4.64	2.4	2.3	1.45	3.57	4.15
Lymphocytes (10^3^/uL)	2.09	2.26	1.99	2.77	1.81	3.35	1.64	2.28	1.68
Vital Signs	Normal	Normal	Normal	Normal	Normal	Normal	Normal	Normal	Normal

Hematology included leukocyte, neutrophil, and lymphocyte counts. Biochemistry results were reviewed for clinically significant abnormalities.

### 3.4 Detection of donor-derived cells in the nasal mucosa

FISH analysis was reported separately from clinical safety outcomes as an exploratory assessment of donor-cell detection in nasal mucosa samples. Nasal swab samples were classified as evaluable or non-assessable according to the presence of sufficient nuclei and adequate hybridization signal. Evaluable FISH samples were available in 4/9 participants before administration, 5/9 participants immediately after administration, and 7/9 participants at Day 7. Among evaluable samples, no Y-chromosome-positive donor-derived cells were detected at any assessed time point. Non-assessable samples were attributable to the scarcity of nuclei or the absence of a hybridization signal. FISH sample evaluability and results are summarized in Tables 6, 7.

**Table 6:** FISH sample evaluability and donor-cell detection.

Time point	Samples collected	Evaluable samples	Non-assessable samples	Y-positive samples among evaluable samples
Pre-administration	9/9	4/9	5/9	0/4
Immediately post-administration	9/9	5/9	4/9	0/5
Day 7 post-administration	9/9	7/9	2/9	0/7

Samples were reported as non-assessable when they have insufficient numbers of preserved/interpretable nuclei or lack of reliable hybridization signal. Donor-derived male cells were not detected above the assay detection threshold in any evaluable sample. However, because several samples were non-assessable, the absence of donor-cell retention cannot be concluded.

**Table 7:** Participant-level FISH sample evaluability and results.

FISH of the “Y” chromosome	1 x 10^6^ cells/administration			5 x 10^6^ cells/administration			10 x 10^6^ cells/administration		
	#1	#2	#3	#4	#5	#6	#7	#8	#9
VR-p	XX	Not assessable	XX	Not assessable	Not assessable	Not assessable	XX	Not assessable	XX
VR-I	XX	XX	Not assessable	Not assessable	Not assessable	Not assessable	XX	XX	XX
VR-7	XX	XX	XX	XX	Not assessable	XX	XX	XX	Not assessable

VR-p, sample prior to administration; VR-I, sample immediately after administration; VR-7, seven days post-administration. XX indicates two X-chromosome signals per nucleus. Not assessable indicates scarcity of nuclei or absence of hybridization signal. No Y-chromosome-positive nuclei were detected in any evaluable sample.

## 4 Discussion

This first-in-human Phase I study evaluated the short-term tolerability and operational feasibility of intranasally administering of third-party, ready-to-use allogeneic CD45RO^+^/RA^−^ memory T lymphocytes derived from a healthy male donor to healthy adult female volunteers. No treatment-emergent adverse events were observed across the tested dose levels, and the highest planned dose, 10 × 10^6^ cells per administration, was successfully delivered to three participants without DLTs during short-term follow-up. Collectively, these findings indicate that intranasal administration was not associated with immediate clinical safety signals under the specific conditions tested, thereby supporting further monitored clinical evaluation.

Administration feasibility was supported by completion of all planned intranasal administrations without dose delays, administration failures, or protocol deviations. In contrast, cell-tracking feasibility was limited, as several nasal swab samples were non-assessable because of insufficient nuclei or an absent hybridization signal.

In the present study, no treated participant reported solicited local nasal symptoms during the on-site observation period or at day seven follow-up visit. Specifically, no nasal pain or discomfort, irritation or burning, rhinorrhea, congestion, sneezing, epistaxis, pruritus, swelling, erythema, or other local reaction was reported. These findings suggest short-term symptom-based local tolerability of intranasal CD45RO^+^/RA^−^ memory T lymphocytes administration under the conditions tested. This observation is particularly relevant given the delicate immunological balance of the nasal mucosa and the potential concern that mucosal immunotherapies might trigger excessive local inflammation. However, local mucosal safety profile was assessed only through directed symptom questioning and clinical observation. Nasal endoscopy, validated mucosal irritation scoring, cytology, biopsy, or imaging were not performed. Therefore, these findings should be interpreted as evidence of short-term symptom-based tolerability rather than as the absence of mucosal inflammation, and subclinical mucosal changes cannot be excluded.

Respiratory viral infections (RSV, Influenza, SARS-CoV-2) cause severe global morbidity and mortality, especially in immunocompromised populations. Current preventative approaches like vaccines and monoclonal antibodies offer limited therapeutic options once severe disease is established (7, 27). Because severe outcomes are driven by maladaptive host immune responses and T-lymphocyte dysregulation rather than viral burden alone (9, 12), restoring cellular immunity is a critical therapeutic target. This Phase I clinical trial evaluates the safety profiel and feasibility of a cellular therapy designed to stimulate and restore effective antiviral cellular immunity in these vulnerable patients.

Intravenous adoptive transfer of third-party, off-the-shelf CD45RO^+^/RA^−^ memory T cells has demonstrated safety and efficacy in severe SARS-CoV-2 and refractory viral diseases due to its low alloreactivity (14–17). This study extends this concept to the intranasal route, directly targeting the respiratory mucosa to provide a non-invasive and feasible therapeutical option. By establishing a localized cellular barrier at the point of viral entry, this approach aims to strengthen upper airway immunity (12, 23). This is particularly critical for young children lacking immunological memory, older adults with senescent T-cell responses (29), and immunocompromised patients who derive limited protection from conventional vaccines (30).

Following intranasal administration, donor-derived memory T cells were not detected in nasal mucosa samples. However, this absence remains inconclusive due to non-assessable swab samples, limited assay sensitivity, and the inability to rule out rapid clearance, tissue migration, or lack of epithelial interaction in non-inflamed tissue. These findings emphasize the necessity of using more sensitive and complementary biodistribution methodologies in future trials. The absence of detectable donor-derived cells may stem from both study design and methodological constraints. Enrolling healthy volunteers lacking mucosal inflammation likely restricted epithelial permeability and cell retention, though transient interactions or downstream regional lymphoid migration cannot be ruled out. Technically, low sample cellularity, suboptimal specimen quality, and the limited individual reliability of FISH tracking likely increased false-negative rates. Nevertheless, these tracking limitations do not compromise the trial's primary objective, which was strictly to establish safety profile and feasibility rather than to prove biodistribution or clinical efficacy.

This study has some limitations. First, the small sample size and short follow-up period (inherent to Phase I trials) limit the detection of rare or delayed adverse events. Although the 7-day safety window was chosen based on the typical 7-to-14-day self-limiting course of respiratory infections (Influenza, RSV, SARS-CoV-2), it is shorter than the standard 28-day DLT window for allogeneic cell therapies. Consequently, delayed alloimmune reactions, cytokine-mediated inflammation, or late hypersensitivity cannot be entirely excluded, despite previous nasal-cell studies supporting the safety of this route in longer periods (31). Second, local tolerability was assessed via clinical interviews, standard monitoring, and laboratory tests, without nasal endoscopy or validated mucosal irritation scales. This was a deliberate protocol decision; the diagnostic yield of such invasive or specialized tools was expected to be limited in these healthy, asymptomatic volunteers. However, subsequent trials in symptomatic patients should incorporate standardized nasal questionnaires and endoscopy to provide more granular local tolerability data.

This first-in-human Phase I study demonstrates that intranasal administration of third-party, off-the-shelf CD45RO^+^/RA^−^ memory T lymphocytes is operationally feasible and was not associated with treatment-emergent adverse events or DTLs during a 7-day follow-up in nine healthy female volunteers. While cell tracking was limited by nasal swab evaluability, these preliminary safety findings represent an important translational milestone. However, they do not confirm long-term safety, cell persistence, or clinical efficacy. Future trials in clinically relevant populations with active infections should incorporate longer follow-up, optimized or repeated dosing, and more sensitive biodistribution and immunological endpoints to assess local immune engagement.

### 4.1 Future directions and next clinical trial

This first-in-human study establishes the safety of intranasal allogeneic CD45RO^+^/RA^−^ memory T lymphocytes up to a cumulative dose of 30x10^6^ cells, providing a framework for future trials. Future clinical development should shift focus toward optimizing mucosal cell retention, sample collection, and cell tracking methods (21, 32).

To maximize therapeutic efficacy, subsequent investigations should explore modified dosing strategies such as repeated administrations and higher cell concentrations. Crucially, future trials should enroll patient populations with active respiratory viral infections or underlying mucosal inflammation. In these patients, the naturally altered epithelial integrity and localized immune activation may facilitate cell retention and functional tissue engagement, unlike the restricted permeability observed in healthy volunteers (9–11).

Overcoming the tracking limitations of standard nasal swabs will require more sensitive, complementary biodistribution methodologies to properly differentiate between rapid clearance and deep tissue migration. Future protocols should evaluate comprehensive collection techniques, such as nasopharyngeal aspirates, to capture all superficial biological material. Additionally, the integration of novel, non-toxic tracking markers like Iron Oxide Nanoparticles warrants consideration, though this approach will require prior validation in preclinical murine biodistribution and toxicity models.

Based on the favorable safety profile demonstrated in this study, progression to a Phase I/II clinical trial is fully justified for patients at high risk of severe respiratory viral disease. In accordance with international clinical development guidelines, the design of this next phase should expand beyond safety to evaluate preliminary signals of biological activity. This will be achieved by pairing standard clinical outcomes with robust, sensitive immunological and viral endpoints.

## Data Availability

The original contributions presented in the study are included in the article/Supplementary material, further inquiries can be directed to the corresponding authors.
