# Supplementary material for: Feasibility and safety profile of intranasal third-party allogeneic CD45RO+/RA− memory T cells: a phase I clinical trial (vRELEASE-I NCT06699758)
Source: Front Med (Lausanne). 2026 Jul 3;13:1832888. doi: 10.3389/fmed.2026.1832888 (PMC13377360; doi:10.3389/fmed.2026.1832888)
Supplement: Supplementary file 1 [file Data_Sheet_1.pdf]

SUPPLEMENTARY TABLES

	HLA-A	HLA-B	HLA-C	HLA-DRB1	HLA-DQB1	HLA-DQA1	HLA MATCHING
**V1**	02:29	44:44	4:16	07:01	02:02	02:02	A*02, DQB1*02
**V2**	02:69	35:44	05:12	04:08	03:04	03:04	A*02, C*05
**V3**	02:03	35:49	04:07	01:01	05:05	01:01	A*02, DQA1*06
**V4**	02:03	14:50	06:08	11:13	03:06	01:05	A*02, DQB1*06, DQA1*01/06
**V5**	03:24	07:07	07:07	01:15	05:06	01:01	DQB1*06, DQA1*01
**V6**	11:24	14:50	06:08	01:07	02:05	01:02	DQB1*02, DQA1*01
**V7**	25:68	18:49	07:07	01:04	03:05	01:03	B*18, DQA1*01
**V8**	23:24	14:44	02:04	01:04	03:05	01:03	DQA1*01
**V9**	02:30	18:35	05:12	03:04	02:03	03:05	A*02/30, B*18, C*05, DQB1*02, DQA1*05
**DONOR**	02:30	18:40	03:05	03:13	02:06	01:05	

Supplementary Table 1 (S1). HLA typing of healthy donor and volunteers (V1 to V9) performed at the Community of Madrid Transfusion Center (Madrid, Spain) on two independent samples by sequence-specific oligonucleotide and next-generation sequencing. In all cases, we found partial HLA-match, with at least one compatible allele.

	Post-depletion, Before freezing (total cells per bag)	After Thawing/washing (total cells, mean)	Viability (%)
Cohort 1 (n=3)	$3x10^{7}$	$1.8x10^{6}$	6,00
Cohort 2 (n=3)	$1.5x10^{8}$	$1.29x10^{7}$	8,62
Cohort 2 (n=3)	3.10^{8}	$2.64x10^{7}$	8,80

Supplementary Table 2 (S2). Cell viability post-depletion/before freezing, and after thawing and washing. In each cohort, three bags were frozen with the cellular product, with an initial quantity of cells of 30 million, 150 million and 300 millions of cells. After thawing and whasing, cells were counted by Trypan-blue staining, obtaining viabilities going to 6% to 9%, reaching the doses aimed for administration.

	Post-Apheresis	Post-depletion, Before freezing
WBC (x10^3/μL)	122.5	19.5
LYM (x10^3/μL)	81.5	10.8
Bag Volume (mL)	120	371
Total lymphocytes	1.47x10^{10}	4.01x10^9
CD45RA$^+$ (%)	29.74	0.01
CD45RO$^+$ (%)	70.26	99.95
Total CD45RA$^+$ cells (x10^3/μL)	4.37x10^9	4.01x10^5
CD45RA$^+$ T cells depleted		4.37x10^9
Total CD45RO$^+$ cells (x10^3/μL)	1.03x10^{10}	4x10^9
CD45RO$^+$ T cells (x10^3/μL) depleted		6.32x10^9
CD45RA$^+$ T cells depletion efficiency (%)		99.99
CD45RO$^+$ T cells enrichment efficiency (%)		38.83

Supplementary Table 3 (S3). Absolute numbers of CD45RA$^+$ and CD45RO$^+$/RA$^-$ cells after apheresis and depletion processes and efficiencies of the procedure. Almost 100% of CD45RA$^+$ cells are eliminated, ensuring the purity of the cellular product that we are administrating.

	Pre-apheresis (%)	Post-apheresis/depletion (%)
Monocytes	29.4	39.1
B cells	15	0.011
Lymphocytes	55.1	51.9
CD45RO$^+$/RA$^-$	39.1	98.4
CD45RO$^+$ NK	0.073	0.031
CD45RO$^+$ CD3$^+$	85.6	87.1
CD45RO$^+$ CD4$^+$	92.8	88.8
CD45RO$^+$ CD8$^+$	1.35	2.11
CD45RA$^+$	46.1	0
CD45RA$^+$ NK	23.1	0
CD45RA$^+$ CD3$^+$	27.9	0
CD45RA$^+$ CD4$^+$	88.2	0
CD45RA$^+$ CD8$^+$	5.64	0

Supplementary Table 4 (S4). Subpopulation percentages of CD45RO$^+$ and CD45RA$^+$ cells, T cells, NK cells, B cells, and monocyte content before apheresis and after apheresis and depletion. Elimination of CD45RA+ cells is almost complete, including NK cells and B cells. Monocytes content is slightly enriched, as others authors previously reported (Teschner et al., 2014. https://doi.org/10.1038/bmt.2013.114).

	IFN-γ response assay (pre-apheresis)			IFN-γ response assay (post- apheresis/depletion/cryopreservation/thawing)		
	% Cells	% IFN-γ^+ (basal)	% IFN-γ^+ (control positive)	% Cells	% IFN-γ^+ (basal)	% IFN-γ^+ (control positive)
**NK cells**	5.56	0.52	18.8	0.057	0	37
**Monocytes**	5.76	1.14	48.3	1.53	3.84	47.2
**Lymphocytes**	55.4			5.36		
**CD45RO$^+$/RA$^-$**	42	0.11	36.4	100	0.26	50.2
**CD45RO$^+$ CD3$^+$**	89	0.13	47.6	95.5	0.18	56.3
**CD45RO$^+$ CD4$^+$**	68	0.11	47	88.5	0.04	55.8
**CD45RO$^+$ CD8$^+$**	26	0.11	55.1	6.07	0	49.7
**CD45RO$^+$ TCM**	83	0.079	56.6	81.5	0.19	54.2
**CD45RO$^+$ TEM**	17	0.3	21.2	18.4	0.56	34.3
**CD4$^+$ TCM**	92.5	0.56	72.6	86.2	0.046	46.3
**CD4$^+$ TEM**	7.24	0.84	74.3	13.6	0	60.7
**CD8$^+$ TCM**	90.5	0.16	71.2	75	0	59.1
**CD8$^+$ TEM**	9.24	1.03	75.3	25	1.52	55
**CD45RA$^+$**	58	0.04	8.73	0		
**CD45RA$^+$ CD3$^+$**	65	0.014	13.4	0		
**CD45RA$^+$ CD4$^+$**	50	0	12.3	0		
**CD45RA$^+$ CD8$^+$**	47	0.014	14.7	0		
**CD45RA$^+$ Naive**	51.4	0.067	21.2	0		
**CD45RA$^+$ TEMRA**	33.7	0.13	13.6	0		
**CD4$^+$ Naive**	89.6	0.3	35.9	0		
**CD4$^+$ TEMRA**	10.4	0.4	66	0		
**CD8$^+$ Naive**	52.3	0.098	22.9	0		
**CD8$^+$ TEMRA**	22	0.83	31.5	0		

Supplementary Table 5 (S5). Donor's PBMCs/CD45RO$^+$RA$^-$ subpopulation percentages and IFN-γ response in a basal condition and after cells stimulation with anti-human CD3 purified plus CD28/CD49d. Cells had an effective IFN-γ response when they are stimulated (positive control) and the subpopulation percentages are within the normal standards and maintained before and after apheresis, depletion, cryopreservation and thawing processes.
